# Metabolic Age as a Potential Marker of Cardiometabolic Vulnerability in Institutionalised Older Adults: A Cross-Sectional Study

**DOI:** 10.3390/jcm15197374

**Published:** 2026-09-23

**Authors:** Noelia Durán-Gómez, Miguel Ángel Martín-Parrilla, Jesús Montanero-Fernández, Casimiro Fermín López-Jurado, Carlos Fernández-Morales, Macarena C. Cáceres

**Affiliations:** 1 Grupo de Investigación Traslacional Biomédica y Sociosanitaria (CTS064), Departamento de Enfermería, Instituto Universitario de Investigación Biosanitario de Extremadura (INUBE), Facultad de Medicina y Ciencias de la Salud, Universidad de Extremadura, 06006 Badajoz, Spain; nduran@unex.es (N.D.-G.); casimirolj@unex.es (C.F.L.-J.); mcaceres@unex.es (M.C.C.); 2 Departamento de Matemáticas, Facultad de Medicina y Ciencias de la Salud, Universidad de Extremadura, 06006 Badajoz, Spain; jmf@unex.es; 3 Departamento de Terapéutica Médico-Quirúrgica, Facultad de Medicina y Ciencias de la Salud, Universidad de Extremadura, 06006 Badajoz, Spain; carlosfm@unex.es

**Keywords:** metabolic age, cardiovascular risk, metabolic syndrome, body composition, institutionalised older adults, geriatric nursing

## Abstract

**Background/Objectives**: Chronological age is a major determinant of cardiovascular risk and metabolic syndrome, but it may not accurately reflect biological ageing or cardiometabolic vulnerability. Metabolic age has emerged as a potential indicator of metabolic health, although its relationship with cardiometabolic risk in institutionalised older adults remains poorly understood. This study examined the association of metabolic age with cardiovascular risk and metabolic syndrome and compared its performance with that of chronological age. **Methods**: A cross-sectional observational study was conducted in residential care facilities in Spain between 2023 and 2024. Fifty-five institutionalised older adults underwent anthropometric and body composition assessment using bioelectrical impedance analysis. Cardiovascular risk was estimated using the Systematic Coronary Risk Evaluation 2–Older Persons (SCORE2-OP) model, and metabolic syndrome was diagnosed according to harmonised clinical criteria. Logistic regression, receiver operating characteristic curve analyses, and discriminant analyses were performed. **Results**: Metabolic age was significantly higher in participants with cardiovascular risk and metabolic syndrome, whereas chronological age showed no significant associations. Metabolic age was significantly associated with both outcomes and showed discriminatory ability within the present sample, whereas chronological age was not significantly associated with either outcome. Leg muscle mass index, body mass index, and visceral fat were the body composition variables most strongly associated with cardiometabolic risk. Higher metabolic age was associated with greater visceral adiposity, and lower leg muscle mass characterised participants with the most adverse cardiometabolic profiles. **Conclusions**: Metabolic age may be a more informative indicator of cardiometabolic vulnerability than chronological age in institutionalised older adults. Combined with body composition assessment, it may provide complementary information for identifying adverse cardiometabolic profiles in ageing populations.

## 1. Introduction

Ageing is a natural physiological process involving biological and genetic pathways, and it is linked to an increased susceptibility to several diseases, including cardiovascular and metabolic disorders [1]. The number of older adults worldwide is rising rapidly. It is estimated that between 2015 and 2050, the proportion of the global population aged over 60 years will nearly double, increasing from 12% to 22% [2]. This highlights the importance of investigating the ageing process and the factors that influence it. Among these factors, cardiovascular disease (CVD) occupies a central role, as it remains one of the leading causes of morbidity and mortality in the ageing population [3] and continues to be the primary cause of death among adults worldwide [4].

Its impact is particularly significant in older individuals, in whom the combination of ageing and metabolic dysfunction exacerbates disease progression. More than 60 million potential years of life are lost annually to CVD in Europe [5]. In this context, several metabolic factors contribute to the development of cardiovascular conditions, with metabolic syndrome (MetS) being a major determinant.

MetS comprises a cluster of metabolic abnormalities, including insulin resistance, atherogenic dyslipidaemia, central obesity, and arterial hypertension. If untreated, MetS is strongly associated with an increased risk of developing both diabetes and CVD [6]. The global prevalence of MetS is approximately 25% [7,8], although estimates range from <10% to 78%, depending on factors such as sex, age, ethnicity, altitude, and even methodological heterogeneity in study design or diagnostic criteria [9,10].

The prevalence of CVD increases with age, and chronological age remains a strong predictor of cardiovascular risk (CVR) [11]. However, chronological age is a non-modifiable risk factor that may not accurately reflect an individual’s health status, given the influence of lifestyle, environmental factors, and comorbidities [12,13]. In addition, other geriatric conditions such as frailty and multimorbidity have been shown to contribute substantially to health vulnerability and poorer physical health outcomes among institutionalised and chronically ill older adults, highlighting the limitations of relying solely on chronological age to characterise health status and risk profiles [14,15]. Indeed, mortality from CVD over the past three decades (1990–2019) has declined not only among younger and middle-aged adults but also in older populations, suggesting that the biological ageing process may be modifiable, even in advanced age [16]. In this regard, metabolic age (Met-age) has been proposed as a potential indicator of metabolic health and may provide complementary information on age-related cardiometabolic vulnerability [17]. Moreover, MetS, which often coexists with cardiovascular disease, is strongly influenced by age-related changes in body composition and metabolic status, thereby reinforcing the need for markers beyond chronological age to capture cardiometabolic risk [18].

Met-age is defined as the comparison between an individual’s basal metabolic rate (BMR) and the average BMR of people within the same chronological age group. A higher BMR corresponds to a lower Met-age, whereas a lower BMR corresponds to a higher Met-age [18]. Met-age thus represents a BIA-derived estimate based on basal metabolic rate and other demographic, anthropometric, and body-composition information incorporated into the device algorithm. A Met-age lower or higher than chronological age indicates how the BIA-derived estimate compares with chronological age; however, its clinical interpretation should be considered in conjunction with established cardiovascular, metabolic, and body-composition measures. Met-age may be influenced by demographic and lifestyle factors. While chronological age and sex are non-modifiable, modifiable factors such as physical activity and adherence to a Mediterranean diet play a substantial role, alongside socioeconomic status and smoking habits [19].

Met-age, along with BMR and other anthropometric and body composition parameters, can be assessed using bioimpedance devices such as TANITA scales [20]. Recent studies have shown that visceral fat, trunk fat percentage, and Met-age are closely correlated with the complexity of coronary artery lesions [21], underscoring the importance of evaluating these parameters in the context of CVD. Few studies, however, have specifically examined the relationship between Met-age, MetS, and CVR. Vásquez-Álvarez et al. [18] reported that Met-age is strongly associated with MetS and may serve as an indicator for individuals at high risk, suggesting that increases in Met-age are associated with greater severity of MetS, independently of chronological age. Similarly, Elguezabal-Rodelo et al., [22] identified a correlation between Met-age and waist-to-height ratio, an established marker of cardiovascular risk.

Considering the physiological changes associated with ageing, it remains unclear whether Met-age or other anthropometric and body composition parameters may better predict the risk of MetS and cardiovascular disease in older adults compared with chronological age. Addressing this knowledge gap is crucial to determine whether Met-age could serve as a more accurate and clinically useful predictor of cardiometabolic risk in ageing populations.

To our knowledge, this is one of the few studies to investigate the relationship between Met-age, CVR, MetS, anthropometric parameters, and body composition in a population of institutionalised older adults. However, some considerations are necessary to distinguish older from younger adult populations, as the participants in this study were elderly. Nevertheless, given the rapid ageing of the population (particularly among individuals with CVD) and the increased nutritional vulnerability of older adults, the results of the present study should be viewed as highly relevant for clinical practice.

In institutionalised older adults, comprehensive biochemical and clinical assessment remains essential for the evaluation of cardiometabolic health. However, repeated blood-based measurements may not always be suitable for frequent monitoring in long-term care settings, particularly given the burden associated with repeated measurements for frail older residents and care staff. In this context, less invasive and continuous monitoring approaches may provide complementary information while reducing the need for frequent conventional measurements [23,24]. In this context, non-invasive, rapid, and easily repeatable tools such as BIA could provide complementary information between more comprehensive clinical assessments. Importantly, Met-age should not be considered a substitute for established biochemical or clinical markers, but rather as a potential adjunctive measure that may facilitate the identification of residents requiring closer cardiometabolic assessment.

The aim of this study was to investigate the association between Met-age, cardiometabolic risk (including CVR and MetS), and anthropometric and body composition parameters in institutionalised older adults, and to compare the associations and discriminatory performance of Met-age with those of chronological age in this high-risk population. Additionally, we sought to explore the potential value of Met-age as a complementary, non-invasive measure of cardiometabolic vulnerability in an institutional care setting.

## 2. Materials and Methods

### 2.1. Design

An observational study with a cross-sectional design and a non-probability sampling approach was conducted in residential and social-health care facilities for older adults in Spain between 2023 and 2024.

This study was reported following the STROBE checklist [25].

### 2.2. Study Setting, Sampling and Selection Criteria

The sample initially consisted of 81 patients. However, 26 patients were lost during the study period. Participants were selected through intentional or convenience non-probabilistic sampling. All patients who remained in the study met the selection criteria, and all participants voluntarily provided informed consent prior to their involvement, thereby upholding ethical standards and respecting the individuals who contributed to the research.

The inclusion criteria required participants to be aged 65 years or older, residing in the selected facilities, and able to provide informed consent (or have a legally authorised representative provide consent in cases of cognitive impairment). Additionally, participants had to have been institutionalised for at least 6 months to ensure stability in their living situation and had to be willing to participate in the study. Exclusion criteria comprised individuals with severe cognitive or physical impairments preventing participation, acute or terminal illnesses, or a history of non-compliance with medical treatment or institutional regulations.

### 2.3. Data Collection

All data collection was conducted face-to-face in the participating nursing homes by a single trained investigator, who remained blinded to the study hypotheses to minimise potential information bias. The researcher was a registered nurse with postgraduate training in health sciences and systematic instruction in the administration of clinical interviews, anthropometric assessments, and study questionnaires, thereby ensuring methodological rigour. To reduce recall bias, questionnaires were administered immediately after the interviews, following standardised written and verbal instructions. Data collection was performed in a quiet and private space within each nursing home, guaranteeing confidentiality and reducing the risk of social desirability bias. The process was closely monitored by the investigator to ensure consistency across participants.

### 2.4. Instruments and Measures

Measurement bias was minimised by employing internationally validated instruments, standardised procedures, and published methods with high reliability. These strategies ensured the robustness and validity of the findings, reinforcing the methodological soundness of the study.

A structured ad hoc sociodemographic questionnaire was developed to collect key participant characteristics, including age, sex, educational level, marital status, employment status, and lifestyle factors. This questionnaire was specifically designed for the study to ensure relevance to the target population and comprehensiveness in data collection. Participants self-reported their responses, with assistance provided when necessary to ensure accuracy.

The medical history of participants was obtained through structured interviews and a review of medical records. Key clinical variables included the presence of chronic conditions (e.g., hypertension, diabetes mellitus, dyslipidaemia, and cardiovascular diseases), current medication use, and previous hospitalizations. Data were cross-validated with clinical documentation to enhance reliability. This information was used to characterise the health status of participants.

Anthropometric parameters and body composition variables, including body-mass index, muscle mass, total body water, visceral fat level, bone mass, and metabolic age and rate, were assessed using the TANITA MC 580M^®^ (TANITA Corporation, Tokyo, Japan), a multi-frequency segmental bioelectrical impedance analyser (BIA). This device employs multiple frequencies to measure impedance across different body segments (trunk, arms, and legs), allowing for detailed evaluation of body composition distribution. Participants stood barefoot on the analyser’s electrodes while holding handgrips, ensuring full-body impedance measurement. The device applied proprietary algorithms to calculate body composition parameters, following standardised manufacturer protocols. Met-age was automatically derived by the TANITA MC-580M using its proprietary algorithm, which relates the estimated basal metabolic rate to age-specific reference values. The estimation of basal metabolic rate and Met-age incorporates demographic, anthropometric, and bioimpedance-derived body-composition information. BIA measurements were performed under standardised pre-analytical conditions following the manufacturer’s recommendations. To minimise variability related to hydration status and body fluid distribution, assessments were performed at a similar time of day and under comparable conditions. Measurements were conducted at least 3 h after the last meal and were avoided immediately after waking, following intense physical activity, or after excessive food or fluid intake. Participants were assessed barefoot, ensuring appropriate contact of the hands and feet with the electrodes, and remained still in a standardised standing position throughout the measurement [26]. Ethical guidelines were strictly followed in data collection and participant handling [27].

To estimate the 10-year risk of fatal and nonfatal cardiovascular events in individuals aged ≥65 years, we employed the Systematic Coronary Risk Evaluation 2–Older Persons (SCORE2-OP) tool. This model incorporates key variables, including age, sex, systolic blood pressure, total and HDL cholesterol levels, smoking status, and the presence of diabetes. It utilises a Cox proportional hazards regression framework to compute individual risk scores and classify individuals into predefined risk categories. As an extension of the SCORE2 model, SCORE2-OP has been specifically recalibrated using region-specific cardiovascular event data to enhance predictive accuracy in older populations. In this study, SCORE2-OP was applied to assess CVR [27].

For the diagnosis of MetS, the harmonised clinical criteria of the American Heart Association were followed [28].

Finally, the difference in age (DIA) was calculated as chronological age minus Met-age [18].

### 2.5. Data Analysis

Data were analysed using SPSS version 29 and R version 4.4.1.

Sample size and sensitivity. No formal a priori sample-size calculation was performed. The sample corresponds to a census of all eligible residents recruited during the predefined study window across participating nursing homes, reflecting the feasibility constraints of this hard-to-reach institutionalised population. To contextualise the precision of the estimates, we report effect sizes and 95% confidence intervals throughout and interpret results cautiously. As a measure of the power corresponding to our sample, we can say that it is sufficient to detect an effect size d = 0.725 (moderate) with a significance level of 5% and a power of 90% for an independent samples *t*-test with the same sample size. Furthermore, it is sufficient to detect an effect size d = 0.365 (weak) with a significance level of 5% and a power of 90% for a paired *t*-test.

A descriptive analysis of the variables was conducted. For inferential analysis, both parametric and non-parametric methods were applied, depending on the distributional symmetry and kurtosis. Specifically, Student’s *t*-test and the Mann–Whitney U test were used to compare two groups; the Chi-square test and Pearson’s correlation coefficient (r) were employed to analyse associations between categorical variables and numerical variables, respectively. Comparisons between correlations were performed using Steiger’s test. Classification problems were addressed through logistic regression, Fisher’s Linear Discriminant Analysis (LDA) with the Wilks’ Lambda selection method, and simple decision trees.

## 3. Results

### 3.1. Characteristics of the Participants

The initial sample consisted of 81 participants, but although many variables could be measured, it was not possible to perform the bioimpedance test due to the functional capacity, balance and coordination of part of the sample. Therefore, a total of 55 participants with an average age of 80.05 ± 9.52 years (range 65–95 years) took part in our study. The excluded subject did not differ significantly from the included subjects in terms of age (*p* = 0.132), sex (*p* = 0.776), or number of pre-existing conditions (*p* = 0.302).

The sociodemographic and clinical variables of the sample are detailed in Table 1. Overall, 65.5% were classified as positive for increased CVR (Table 1) and 54.5% met the MetS requirements. Females made up 76.4% of the sample, but there was no difference in the distribution between the CVR(−) group and the CVR(+) group. The same was true for the MetS(+) and MetS(−) groups. As anticipated, the CVR(+) and MetS(+) groups showed older age, but these differences were not significant. Met-age in the CVR(+) group was significantly higher than in the CVR(−) group (*p* < 0.001) (Table 1 and Figure 1). Likewise, the Met-age for the MetS(+) group was notably higher compared to the MetS(−) group (*p* < 0.001) (Table 1 and Figure 1).

These findings indicate that higher Met-age values were associated with CVR and MetS classifications in this sample. However, to account for the difference between chronological age and Met-age, we calculated their DIA. In this case, as the population was older than young adults, mean DIA values for each group were positive. However, it was observed that the CVR(+) and MetS(+) groups had Met-age values closer to their chronological age compared to those without. In fact, significant differences were found for both CVR and MetS classifications (*p* = 0.044; *p* = 0.001).

A weak association was observed between Met-age and chronological age (r = 0.293, *p* = 0.030).

Additionally, nutritional status was assessed using the Mini Nutritional Assessment (MNA) to explore its potential relationship with cardiometabolic vulnerability. No statistically significant associations were observed between MNA scores and CVR (*p* = 0.105) or between MNA scores and Met-age (*p* = 0.170).

### 3.2. Association Between Anthropometric Parameters and Body Composition with Met-Age, Chronological Age, CVR and MetS

As for body composition measures, using simple correlations, we assessed the effect of body-mass index (BMI), body fat, muscle mass, bone mass, body water, visceral fat and leg muscle mass index with Met-age (Table 2). These parameters influence Met-age; therefore, the relationship between body composition and Met-age or chronological age was analysed. The interpretation of the results is as follows.

Correlations with chronological age (Table 2):

Body fat percentage: A negative weak correlation was observed (r = −0.287, *p* = 0.034), indicating that higher chronological age was slightly associated with lower body fat.

Body water: A weak negative correlation was found (r = −0.324, *p* = 0.016), suggesting a small decline in body water with age.

Visceral fat: A weak negative correlation (r = −0.290, *p* = 0.031) indicated a slight decrease in visceral fat with increasing age.

Correlations with Met-age (Table 2):

BMI: A moderate positive correlation (r = 0.516, *p* < 0.001) indicated that higher BMI was associated with higher Met-age.

Body fat percentage: A moderate-to-strong positive correlation (r = 0.574, *p* < 0.001) suggested that increased fat percentage was strongly linked to higher Met-age.

Visceral fat: A strong positive correlation (r = 0.581, *p* < 0.001) demonstrated that greater visceral fat was associated with higher Met-age.

Leg muscle mass index: A strong negative correlation (r = −0.644, *p* < 0.001) showed that greater leg muscle mass was strongly related to lower Met-age.

All differences in correlation signs were confirmed by Steiger’s test. Taken together, these results indicate that chronological age showed only weak correlations with the analysed variables, whereas Met-age showed moderate to strong associations with BMI, body fat, visceral fat, and leg muscle mass index. Taken together, Met-age showed moderate-to-strong associations with several body-composition parameters, whereas chronological age showed only weak relationships. However, because Met-age is itself derived from an algorithm incorporating anthropometric and bioimpedance-derived information, these correlations are subject to mathematical coupling and should therefore be interpreted as descriptive associations rather than as evidence that Met-age provides independent information beyond its constituent measures.

When comparing the CVR(–) and CVR(+) groups in terms of anthropometric and body composition variables (Table 1), BMI was significantly higher in CVR(+) (*p* = 0.023, d = −0.666), reflecting greater weight accumulation relative to height in this group. Body fat was also significantly higher in CVR(+) (*p* = 0.013, d = −0.731), indicating that this group has higher levels of body fat. Muscle mass was higher in CVR(+) (*p* = 0.029, d = −0.638), possibly due to greater overall body weight. Bone mass was slightly higher in CVR(+) (*p* = 0.038, d = −0.605), although with a small effect size. Likewise, the CVR(+) group has a significantly higher percentage of water (*p* = 0.012, d = −0.736), possibly related to the higher weight and muscle mass. Visceral fat was significantly higher in CVR(+) (*p* = 0.006, d = −0.805), indicating an elevated cardiovascular and metabolic risk. In contrast, leg muscle mass index was significantly lower in CVR(+) (*p* < 0.001, d = 0.992), suggesting a reduction in specific muscle mass in the lower limbs. Considering the metabolic rate, this was significantly lower in CVR(+) (*p* = 0.003, d = 0.900), indicating a reduced metabolism in this group.

These results suggest that the CVR(+) group shows a body composition profile associated with higher metabolic and cardiovascular risk. Effect sizes were moderate to large, highlighting the importance of these differences in key variables such as visceral fat (d = −0.805), metabolic rate (d = 0.900) and leg muscle mass index (d = 0.992).

When studying the MetS(+) and MetS(−) groups, we also found significant differences in body composition and anthropometric measures (Table 1). BMI was significantly higher in MetS(+) (*p* < 0.001, d = −1.315), reflecting a notable accumulation of weight in relation to height in this group. The MetS(+) group exhibited significantly higher body fat (*p* < 0.001, d = −1.075), reinforcing the obesity profile linked to this syndrome. Muscle mass was significantly higher in MetS(+) (*p* = 0.032, d = −0.597), which may reflect the higher body weight of this group. Bone mass was also significantly higher in MetS(+) (*p* = 0.040, d = −0.571), although with a low effect size. Body water percentage was higher in MetS(+) (*p* = 0.003, d = −0.838), probably associated with the increase in total body mass. Visceral fat was significantly higher in MetS(+) (*p* < 0.001, d = −1.309), showing a clear marker of metabolic risk in this group. The MetS(+) group has significantly less muscle mass index in the legs (*p* < 0.001, d = 1.121), suggesting a specific impairment in the peripheral musculature. Finally, the metabolic rate was significantly lower in MetS(+) (*p* < 0.001, d = 1.224), indicating a slower metabolism in this group.

These results suggest that MetS(+) is associated with a distinctly unfavourable body composition profile. Effect sizes were particularly large for key variables such as visceral fat (d = −1.309), BMI (d = −1.315), leg muscle mass index (d = 1.121), and metabolic rate (d = 1.224), indicating marked differences between the groups.

### 3.3. Association and Discriminatory Performance of Met-Age for CVR and MetS

To examine the relationship of chronological age and metabolic age with CVR and MetS, two multiple logistic regression models were fitted. In the first model, CVR was considered the outcome variable, whereas in the second model MetS was considered the outcome variable; chronological age and Met-age were included as explanatory variables in both analyses. Receiver operating characteristic (ROC) curves were used to evaluate the ability of both age measures to discriminate between participants with and without CVR or MetS (Figure 2). Given the small sample size, confidence intervals were estimated using bias-corrected bootstrap resampling with 1000 replicates to obtain more reliable estimates.

For CVR, chronological age was not significantly associated (OR = 0.990, 95% bias-corrected CI: 0.918–1.068, *p* = 0.821), whereas Met-age showed a significant association (OR = 1.122, 95% bias-corrected CI: 1.030–5.124, *p* = 0.002), with an AUC of 0.728 (Figure 2a). Similarly, for MetS, chronological age was not statistically significant (OR = 0.917, 95% bias-corrected CI: 0.739–0.978, *p* = 0.064), while Met-age demonstrated a significant association (OR = 1.163, 95% bias-corrected CI: 1.051–2.259, *p* < 0.001), with an AUC of 0.782 (Figure 2b). These findings indicate that Met-age was more strongly associated with both CVR and MetS than chronological age and showed discriminatory ability for both classifications within the present sample.

The relationship between CVR and MetS was weak, as 76.7% of subjects with MetS(+) were also CVR(+), and 48.0% of those with MetS(–) were CVR(–). This association was of borderline significance (*p* = 0.055).

### 3.4. Anthropometric Parameters and Body Composition in Relation to CVR and MetS

Given the sample size and the moderate to strong correlations observed between anthropometric parameters and body composition (Table 2), it was not possible to clearly determine which parameters showed the strongest discriminatory contribution to CVR and MetS classifications. However, Wilks’ Lambda algorithm was used as a criterion in the Linear Discriminant Analysis (LDA) to select the most appropriate parameters based on the data. First, when CVR was considered as the outcome, leg muscle mass index was selected (r = 0.433, *p* = 0.001). Second, when MetS was considered as the outcome, BMI and visceral fat were selected (R = 0.600, *p* < 0.001 for both).

Figure 3, Figure 4 and Figure 5 summarises the influence of chronological age, Met-age, anthropometric parameters, and body composition on CVR and MetS. For clearer visualisation, the Y-axis represents the Discriminant Index Algorithm. Therefore, the horizontal lines at zero distinguish participants whose Met-age was higher than their chronological age (above the line) from those whose Met-age was equal to or lower than their chronological age (below the line). The three previously selected parameters are represented on the X-axis: leg muscle mass in Figure 3, visceral fat in Figure 4, and BMI in Figure 5. In each figure, red dots represent participants with CVR in the upper panel and those with MetS in the lower panel. All participants whose Met-age exceeded their chronological age had either CVR or MetS, and most had both conditions.

The vertical dashed lines indicate the cut-off values identified by the decision tree. These thresholds should not be interpreted in terms of statistical inference; rather, they represent data-driven cut-off points that optimally discriminated, within our sample, participants with CVR or MetS (red) from those without these conditions (green). The resulting thresholds were 88 for leg muscle mass, 0.50 for visceral fat, and 29.1 for BMI.

Regarding leg muscle mass index (Figure 3), most participants to the right of the dashed line, corresponding to higher leg muscle mass, were healthy and had a Met-age lower than their chronological age. A similar trend was observed for BMI (Figure 4 and Figure 5, respectively) and visceral fat, although in this case for participants to the left of the dashed line.

## 4. Discussion

This study provides novel evidence on the relationship between Met-age, CVR, MetS, and body composition in institutionalised older adults. Our findings revealed that Met-age was significantly higher in participants with both CVR(+) and MetS(+) compared with their counterparts, while chronological age was not significantly associated with either classification. Logistic regression analyses further indicated stronger associations of Met-age with CVR and MetS than chronological age, while ROC analyses showed the discriminatory ability of Met-age for both classifications within the present sample. Importantly, Met-age showed moderate to strong correlations with BMI, body fat, visceral fat, and leg muscle mass index, whereas chronological age exhibited only weak associations with these parameters. This suggests that Met-age may reflect body composition and metabolic characteristics that are not captured by chronological age alone in this population. However, these associations require cautious interpretation because Met-age is derived from estimated basal metabolic rate and incorporates anthropometric and bioimpedance-derived body-composition information. Consequently, its correlations with BMI, body fat, visceral fat, and muscle-related variables are not statistically independent, and part of their observed strength may reflect mathematical coupling resulting from shared components in their derivation. Accordingly, these correlations should be interpreted as descriptive associations rather than as independent validation of Met-age or evidence that Met-age is intrinsically superior to the individual body-composition measures.

### 4.1. Association Between Anthropometric Parameters and Body Composition with Met-Age, Chronological Age, CVR and MetS

Our study shows that Met-age was more closely associated with anthropometric and body composition parameters than chronological age. In particular, Met-age exhibited moderate to strong correlations with BMI, visceral fat, body fat percentage, and leg muscle mass index, whereas chronological age displayed only weak associations. These findings suggest that Met-age integrates multiple physiological changes related to ageing, capturing both adiposity and muscle mass status, and may therefore provide complementary information on cardiometabolic vulnerability beyond chronological age.

Previous evidence supports this interpretation. Vásquez-Álvarez et al., [18] demonstrated that higher Met-age was strongly associated with MetS, independently of chronological age, while Elguezabal-Rodelo et al., [22] found stronger correlations between Met-age and waist-to-height ratio compared with chronological age. More recently, Wang et al. showed that a lipidomic-based Met-age score predicted cardiometabolic disease independently of chronological age, reinforcing the concept that biological ageing is a better determinant of metabolic health [17]. In contrast, studies such as Hamczyk et al., [12] and the SCORE2-OP models [29] highlighted the robust predictive value of chronological age at the population level. These apparent discrepancies may be explained by differences in methodology and study populations: large cohorts typically include community-dwelling adults, while our sample involved institutionalised older adults with distinct metabolic and nutritional vulnerability.

Our findings also align with the growing recognition that BMI alone is an insufficient marker of cardiometabolic health. Lang et al. demonstrated the heterogeneity of BMI, showing that fat and lean mass contribute differently to health outcomes [30]. Similarly, Blüher [31] concluded that the so-called ‘metabolically healthy obesity’ is largely a transient state, with most individuals eventually developing increased cardiometabolic risk. Taken together, these studies reinforce the idea that Met-age, which reflects both adiposity and lean mass, may provide complementary information on metabolic and body composition status. This interpretation is further supported by reviews on biological ageing, which stress that molecular and systemic ageing processes—rather than chronological age per se—drive cardiovascular vulnerability [1,16].

Taken together, these findings provide preliminary evidence from institutionalised older adults showing that Met-age was more strongly associated with several anthropometric and body composition parameters than chronological age. This suggests that Met-age may provide complementary information on cardiometabolic vulnerability in this specific population.

### 4.2. Association and Discriminatory Performance of Met-Age for CVR and MetS

Regression models showed that Met-age was significantly associated with both CVR and MetS classifications, whereas chronological age was not. ROC analyses showed AUC values of 0.728 for CVR and 0.782 for MetS, indicating discriminatory ability within the present sample. In the logistic regression models, each additional year of Met-age was associated with higher odds of being classified as CVR(+) and MetS(+).

It should also be considered that CVR, as classified using SCORE2-OP, and MetS are not entirely independent constructs, as both reflect overlapping components of cardiometabolic health [27]. Nevertheless, in our sample, the association between CVR and MetS classifications was weak and did not reach statistical significance (*p* = 0.055), indicating that the two classifications were not interchangeable. Therefore, the association of Met-age with both outcomes should not be interpreted as evidence of two completely independent relationships, but rather as reflecting its association with partially overlapping dimensions of cardiometabolic vulnerability. This overlap may also contribute to the observed associations between Met-age and both classifications, given that Met-age itself reflects metabolic and body-composition characteristics.

Met-age was also closely associated with multiple aspects of body composition, including both adiposity and muscle mass status. However, these associations require cautious interpretation because Met-age is derived from estimated basal metabolic rate and incorporates anthropometric and bioimpedance-derived body-composition information. Consequently, its correlations with BMI, body fat, visceral fat, and muscle-related variables are not statistically independent, and part of the observed strength of these associations may reflect mathematical coupling resulting from shared components in their derivation. Accordingly, these correlations should be interpreted as descriptive associations rather than as independent validation of Met-age or evidence that Met-age is intrinsically superior to the individual body-composition measures.

These findings are consistent with international epidemiological evidence. The PURE cohort demonstrated substantial variability in CVD incidence across 21 countries, which cannot be fully explained by traditional risk factors, highlighting the need for refined predictive tools [4]. The World Health Organization cardiovascular disease risk charts provide pragmatic models for global use, including laboratory and non-laboratory formats, but they rely heavily on chronological age [11]. Mazalin Protulipac et al. [32], in a large study of adults aged >70 years, also reported a discrepancy between objectively assessed cardiovascular risk factors and individuals’ perceptions of their own risk, highlighting the importance of objective cardiovascular risk assessment in older populations. In European populations, CVD continues to be the leading cause of mortality, accounting for millions of years of life lost [5]. In this context, our findings suggest that Met-age may provide complementary information on cardiometabolic vulnerability alongside chronological age and established risk-assessment approaches. However, its incremental clinical value and prospective predictive validity require confirmation in larger, prospective studies.

Mechanistically, our findings are supported by evidence that central adiposity and ectopic fat are central drivers of cardiometabolic risk. Neeland et al. [8] concluded that visceral adiposity is a key determinant of MetS and cardiometabolic disease, consistent with our observation that visceral fat was among the strongest discriminators of CVR and MetS. By reflecting both excess adiposity and reduced muscle mass, Met-age likely integrates the biological pathways underpinning cardiovascular and metabolic ageing.

### 4.3. Anthropometric Parameters and Body Composition in Relation to CVR and MetS

Discriminant analysis identified leg muscle mass index, BMI, and visceral fat as the variables that contributed most strongly to discrimination according to cardiometabolic risk status. Participants with CVR(+) and MetS(+) presented an unfavourable phenotype characterised by higher Met-age, greater visceral adiposity, lower leg muscle mass, and reduced basal metabolic rate. Effect sizes for these parameters were moderate to large, indicating their clinical relevance.

This pattern is consistent with the concept of sarcopenia and, more specifically, sarcopenic obesity, defined by the coexistence of muscle loss and excess adiposity. Sarcopenia is recognised as a major determinant of frailty and adverse health outcomes in older adults [33]. Lee et al., [34] and Kim et al., [35] demonstrated that reduced appendicular muscle mass, particularly in the lower limbs, is associated with insulin resistance and higher prevalence of MetS. More recently, Seko et al., [36] showed that preserved lower limb muscle mass protects against insulin resistance, independent of overall obesity. Our findings echo this evidence, as participants with reduced leg muscle mass and higher visceral adiposity were those with the highest cardiometabolic risk.

Large-scale longitudinal studies reinforce these associations. Jiang et al. [37], using the CHARLS cohort, reported that sarcopenic obesity increased cardiovascular disease risk by 39%, while Farmer et al. [38], analysing the UK Biobank, found that obesity and low grip strength were independently associated with higher cardiovascular and all-cause mortality. Additionally, Janssen et al., [39] highlighted the importance of body composition balance, showing that a greater proportion of lean mass relative to fat mass was linked to a more favourable cardiometabolic profile. Gradidge et al., [40] also found that central adiposity and total fat mass were strongly associated with MetS in women with high cardiometabolic risk.

Mechanistically, visceral fat is a metabolically active tissue with endocrine and pro-inflammatory properties. It contributes to insulin resistance, systemic inflammation, and pro-thrombotic states, all of which underpin the development of MetS and CVD [41,42,43,44]. Recent evidence further indicates that visceral adipose tissue is independently associated with residual cardiovascular risk, even after controlling for traditional risk factors [44]. This reinforces the concept that visceral adiposity plays a central role in cardiometabolic vulnerability and supports our observation that participants with higher visceral fat showed greater CVR and MetS.

Although the present analysis focused on the amount of visceral fat as assessed by bioimpedance, other evidence suggests that qualitative aspects of adipose tissue may also play an important role. Lee et al. [45] demonstrated that lower abdominal fat density, measured by CT attenuation, was associated with adverse adipokine profiles, independent of total fat volume. Future studies should therefore consider both the quantity and quality of adipose tissue when evaluating cardiometabolic vulnerability in older adults.

### 4.4. Strength and Limitations of the Work

This study has several limitations. Its cross-sectional design precludes causal inference. The relatively small sample size and the inclusion of institutionalised older adults from a single region limit the generalisability of the findings. Although limited in size, the sample represented a census of eligible residents during the recruitment period. Twenty-six participants were excluded because they were unable to undergo the BIA assessment due to their functional condition. This represents a potential source of selection bias, as individuals with poorer functional status may be underrepresented in the final sample. Consequently, the findings should not be generalised to the most functionally impaired segment of the institutionalised older population. Body composition was assessed using bioimpedance analysis, which, despite being widely used in clinical practice, is less accurate than gold-standard techniques such as dual-energy X-ray absorptiometry. Furthermore, although BIA measurements were performed under standardised pre-analytical conditions to minimise variability related to hydration and body fluid distribution, hydration status was not independently quantified using specific biomarkers. Therefore, the potential influence of subclinical dehydration, peripheral oedema, or other alterations in body fluid distribution on BIA-derived estimates cannot be excluded, particularly in an institutionalised older population. An additional methodological limitation concerns the proprietary derivation of Met-age. Because its calculation incorporates anthropometric and bioimpedance-derived body-composition information, correlations with variables such as BMI, body fat, visceral fat, and muscle-related measures are subject to mathematical coupling and may therefore be inflated. These associations should not be interpreted as independent validation of Met-age. Moreover, because the complete TANITA algorithm, including the exact mathematical formulation and weighting of its components, is proprietary, the extent of this mathematical dependence cannot be quantified. In addition, although nutritional status was explored using the MNA, other potentially relevant confounding variables, including detailed dietary intake, physical activity and mobility, specific medication use, and inflammatory biomarkers, were not incorporated into the regression models and may have influenced the observed associations. Accordingly, the observed associations should not be interpreted as independent effects of Met-age beyond all established clinical and biochemical cardiometabolic risk factors.

Despite these limitations, this study provides novel evidence of associations between Met-age, cardiovascular risk, metabolic syndrome, and adverse body-composition profiles in institutionalised older adults, while chronological age was not significantly associated with CVR or MetS classifications in the present sample. The inclusion of body composition measures alongside Met-age assessment represents a relevant strength, as it enabled the identification of phenotypic characteristics linked to increased cardiometabolic vulnerability in this high-risk population.

### 4.5. Recommendations for Further Research

Future research should validate these findings in larger, multicentre, and longitudinal cohorts of institutionalised and community-dwelling older adults. Longitudinal studies are particularly needed to determine whether metabolic age predicts incident cardiovascular events, metabolic syndrome progression, frailty, disability, and mortality over time. In addition, future investigations should evaluate whether integrating metabolic age into existing cardiovascular risk prediction models improves their predictive accuracy and clinical utility in older populations.

Further studies should also explore the biological mechanisms linking metabolic age, visceral adiposity, sarcopenia, and cardiometabolic vulnerability. The inclusion of inflammatory biomarkers, nutritional status, physical activity, and functional capacity measures may help clarify the pathways underlying these associations. Finally, intervention studies assessing the effects of exercise, nutritional strategies, and multidisciplinary lifestyle programmes on metabolic age and body composition could provide evidence for targeted prevention and management approaches in vulnerable ageing populations.

### 4.6. Implications for Policy and Practice

The potential clinical relevance of these findings should be considered from a complementary rather than substitutive perspective. Comprehensive clinical and biochemical assessment remains essential for the evaluation of cardiometabolic health, and Met-age cannot replace established cardiovascular risk factors, laboratory biomarkers, or medical assessment. However, repeated comprehensive evaluations may be difficult to perform frequently in institutionalised older adults because of resource requirements and the additional burden that invasive procedures may impose on frail residents. In this context, BIA offers a rapid, non-invasive, and easily repeatable assessment that can be performed at the point of care. Met-age and the accompanying body composition measures could therefore provide complementary information during routine monitoring and potentially help identify residents in whom a more comprehensive clinical or biochemical assessment may be warranted.

From a nursing perspective, regular monitoring of visceral adiposity, muscle mass, and metabolic age may help nurses identify adverse functional and metabolic profiles, guide tailored care plans, and promote interventions aimed at preserving mobility, nutritional status, and cardiometabolic health. Multidisciplinary care pathways integrating nursing assessment, nutritional support, and physical activity programmes may be particularly beneficial in institutionalised settings.

At the policy level, these preliminary findings support further investigation of accessible body composition measures in long-term care settings. If confirmed in larger prospective studies, BIA-derived measures could potentially complement established approaches to cardiometabolic assessment and contribute to preventive and person-centred models of care.

## 5. Conclusions

Met-age was more strongly associated with CVR and MetS than chronological age in institutionalised older adults and demonstrated discriminatory ability for identifying individuals with adverse cardiometabolic profiles within the present sample. Met-age was also associated with key body composition characteristics, particularly visceral fat, BMI, and leg muscle mass index, suggesting that it may provide complementary information on metabolic and body composition status beyond chronological age alone. These findings suggest that bioimpedance-derived Met-age may provide complementary information on cardiometabolic vulnerability in institutionalised older adults.

Importantly, Met-age is not proposed as a substitute for established biochemical, cardiovascular, or clinical assessment. Rather, its potential value lies in providing a rapid, non-invasive, and easily repeatable adjunctive measure that may be particularly useful for routine monitoring in long-term care settings. Given the cross-sectional design, limited sample size, and use of a proprietary bioimpedance-derived measure, these findings should be considered preliminary and hypothesis-generating. Larger multicentre and longitudinal studies are required to determine whether Met-age provides incremental clinical value beyond established cardiometabolic risk factors and biochemical markers.

## Figures and Tables

**Figure 1:**
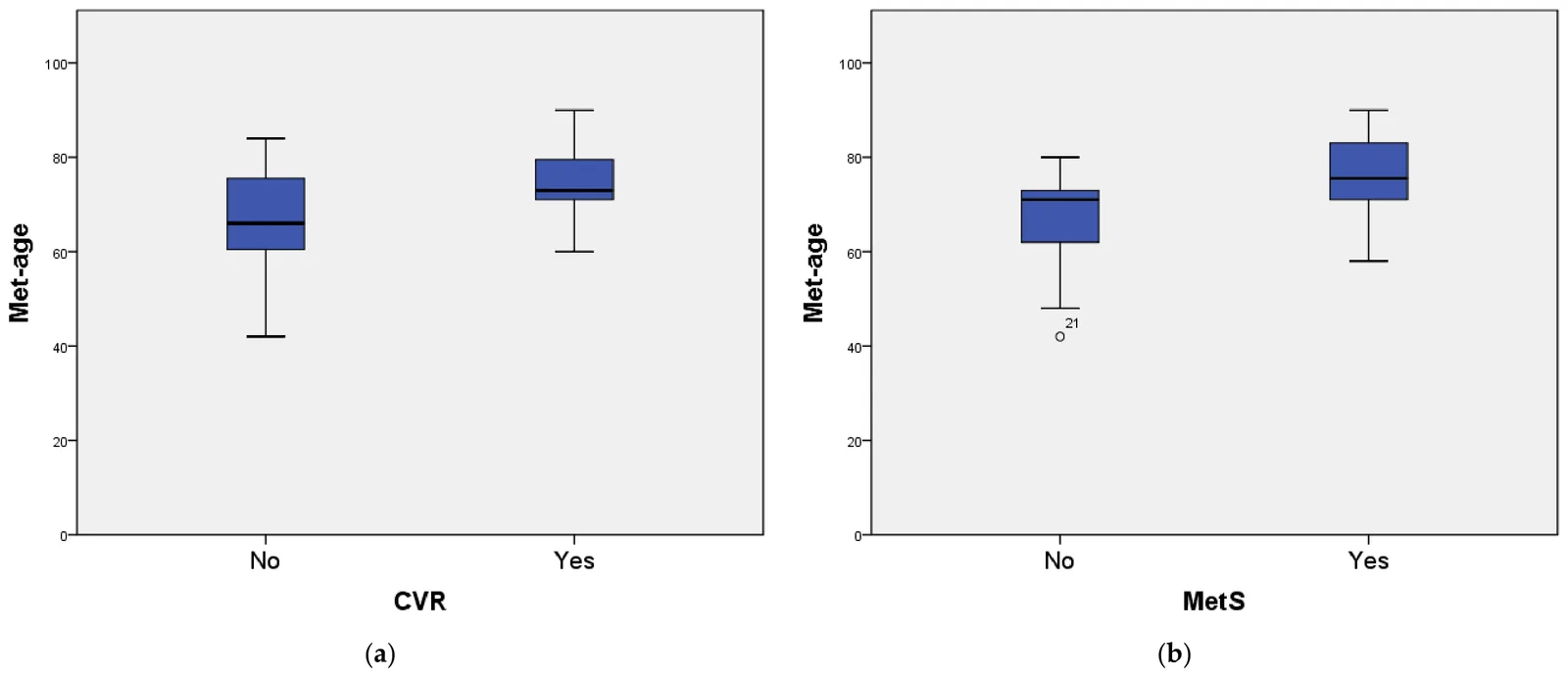
Relationship between metabolic age (Met-age), (**a**) CVR and (**b**) MetS.

**Figure 2:**
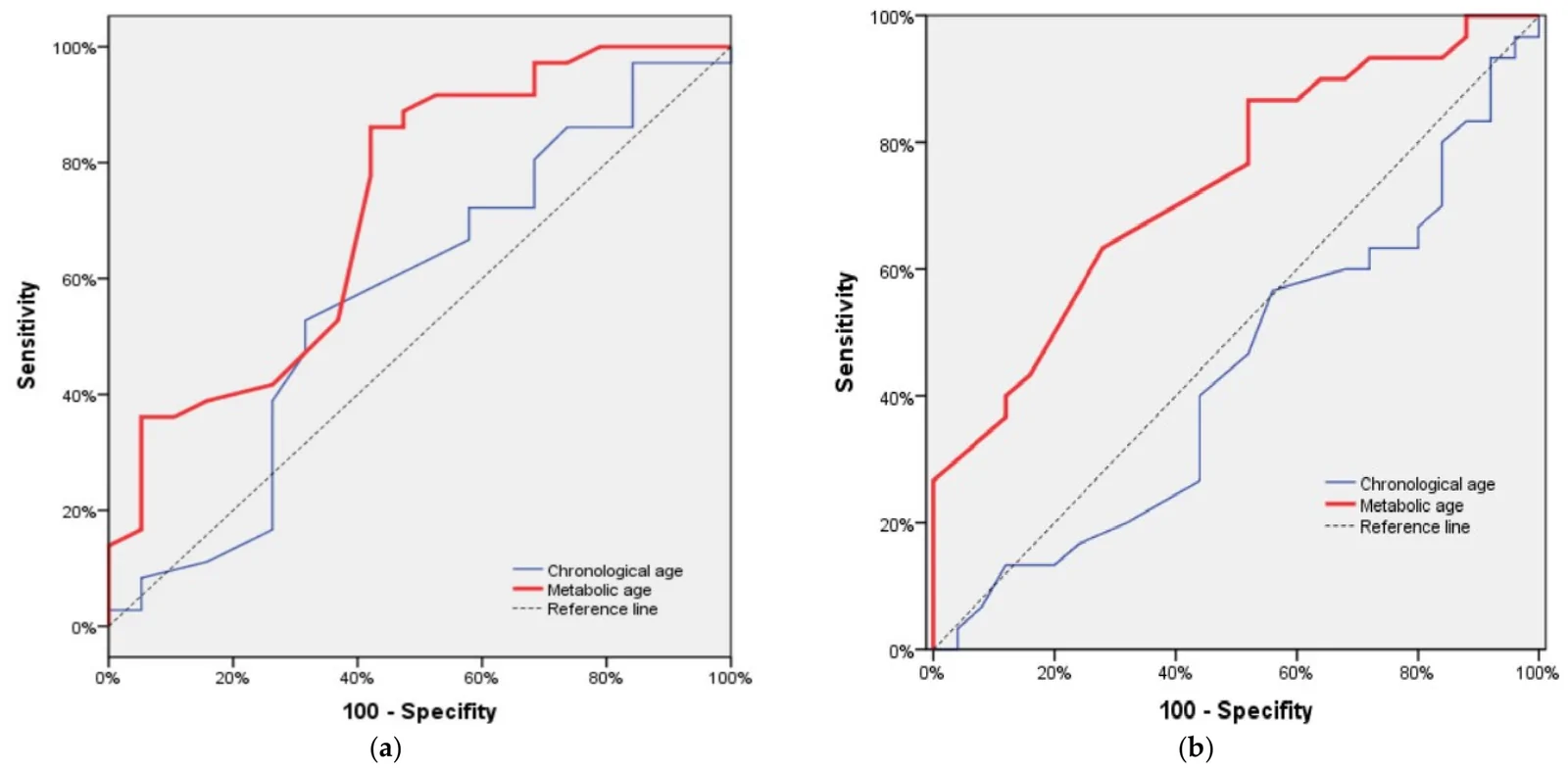
Discriminatory performance of Met-age and chronological age for CVR and MetS classifications. ROC curves were constructed, and the AUC was calculated to assess the ability of Met-age and chronological age to discriminate between participants with and without (**a**) CVR and (**b**) MetS.

**Figure 3:**
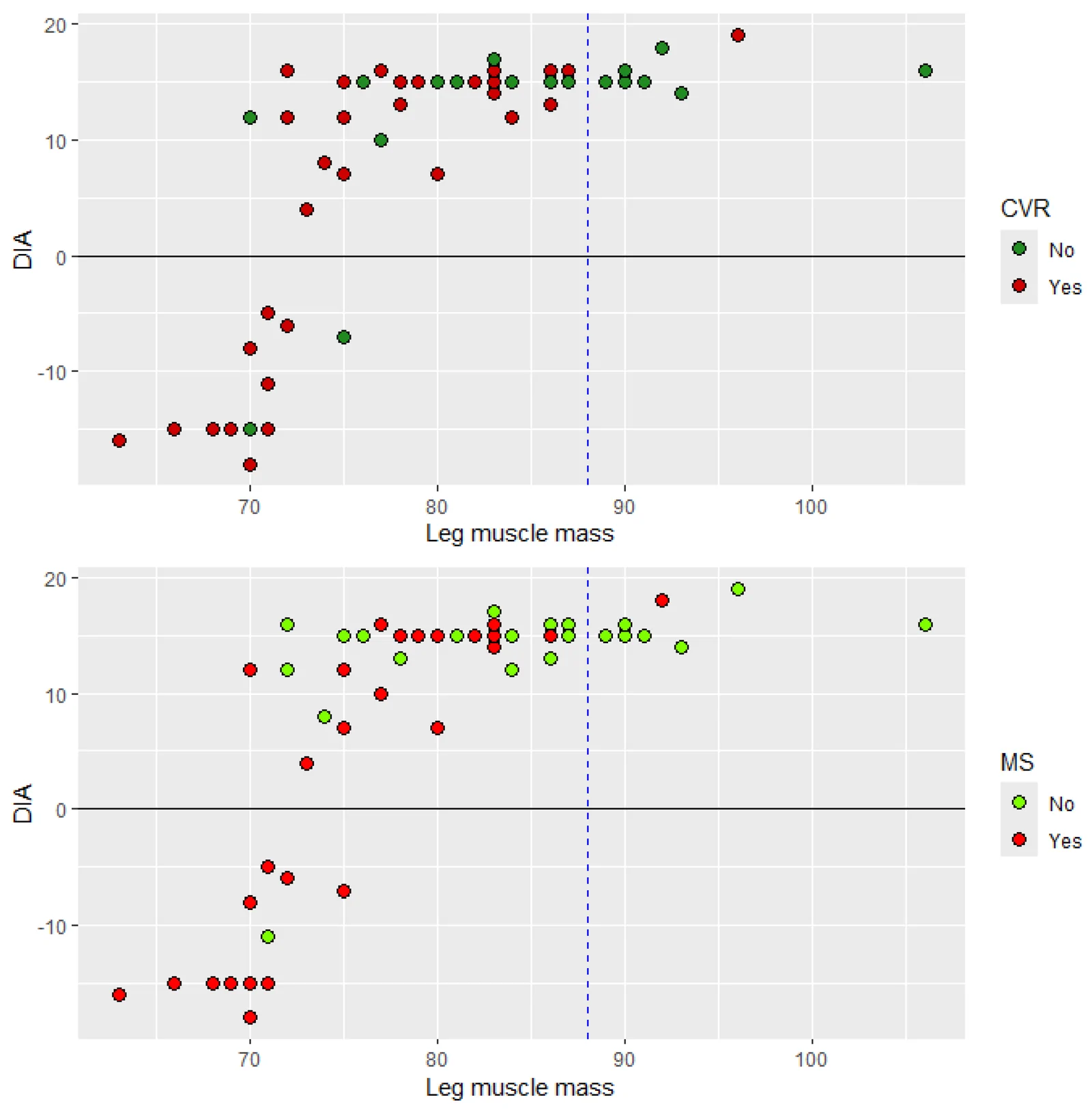
Relationship between difference in age (DIA) and leg muscle mass index according to CVR status (**upper** panel) and MetS status (**lower** panel). The vertical blue line represents a threshold determined by a decision tree to distinguish subjects with CVR or MetS from the rest, based on leg muscle mass.

**Figure 4:**
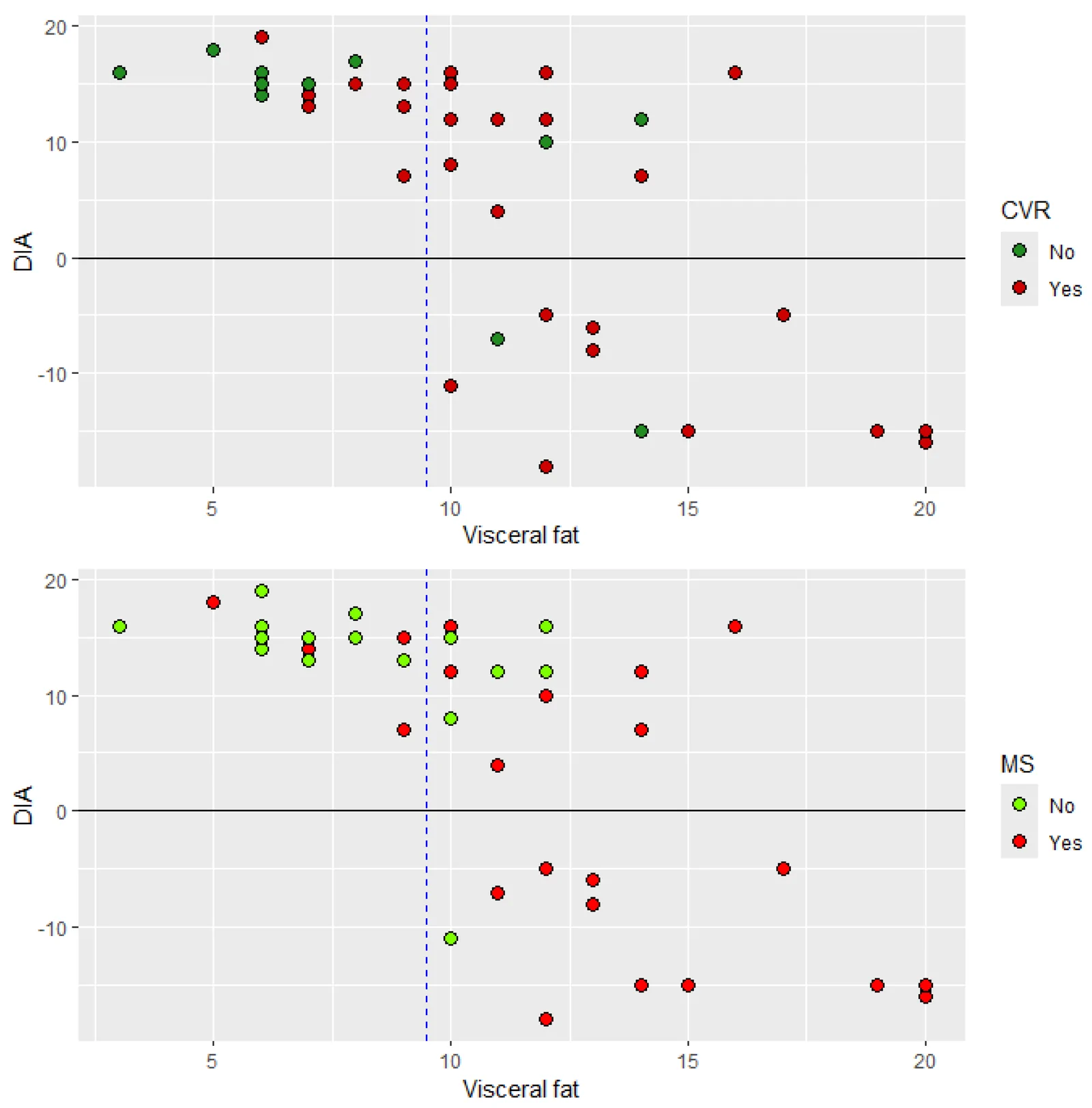
Relationship between DIA and visceral fat according to CVR status (**upper** panel) and MetS status (**lower** panel). The vertical blue line represents a threshold determined by a decision tree to distinguish subjects with CVR or MetS from the rest, based on visceral fat.

**Figure 5:**
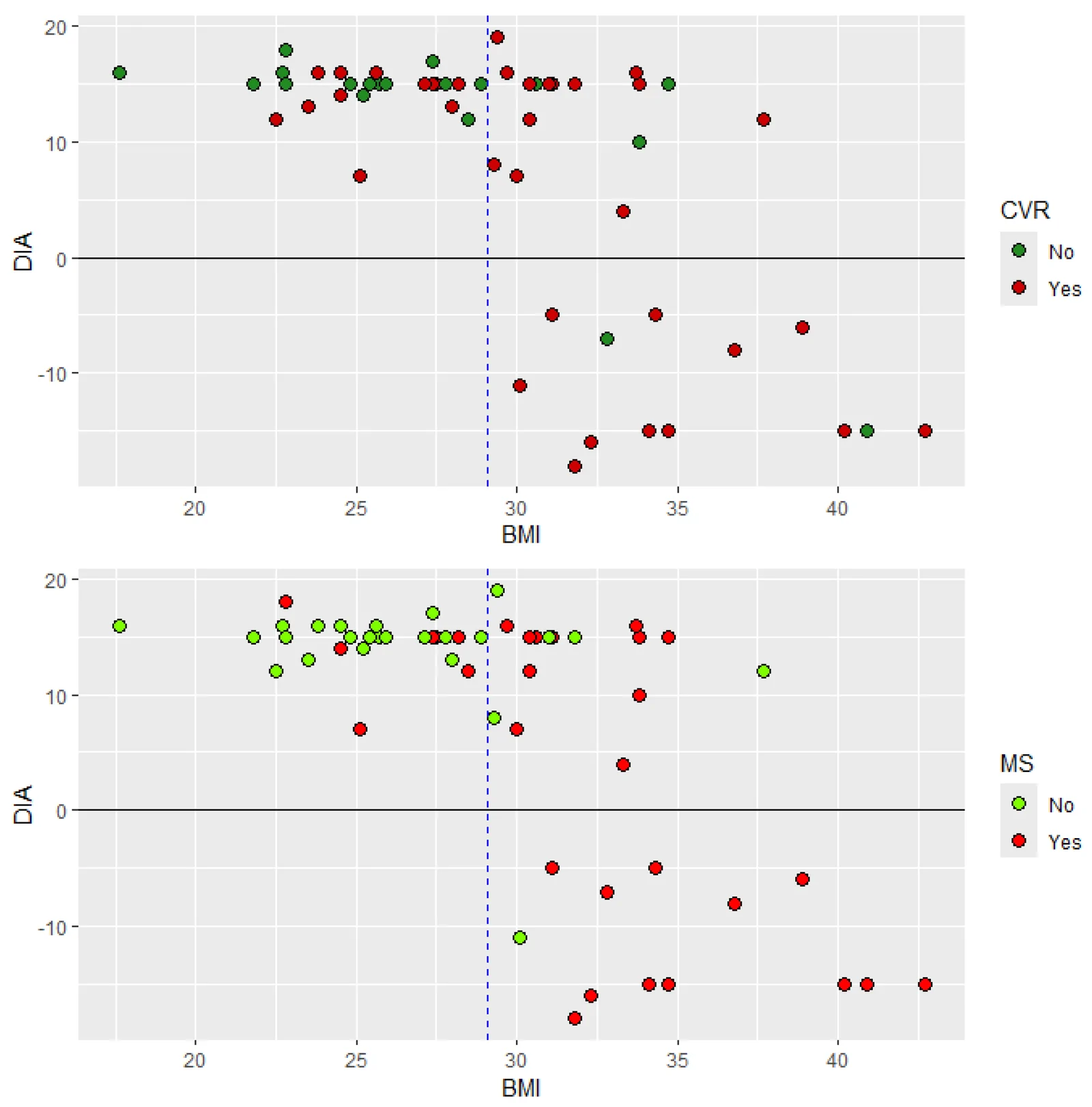
Relationship between DIA and body-mass index according to CVR status (**upper** panel) and MetS status (**lower** panel). The vertical blue line represents a threshold determined by a decision tree to distinguish subjects with CVR or MetS from the rest, based on BMI.

**Table 1:** Clinical, anthropometric, and body composition variables in institutionalised older adults by cardiovascular risk (CVR) and metabolic syndrome (MetS) categories.

Category	CVR (−)	CVR (+)	Sig.	Cohen’s d	MetS (−)	MetS (+)	Sig.	Cohen’s d
Gender (n) ^a^	19	36	0.096		25	30	0.562	
Male	2	11			5	8		
Female	17	25			20	22		
Age (years) ^b^	78.579 ± 10.521	80.833 ± 9.006	0.409	−0.236	81.120 ± 9.248	79.167 ± 9.809	0.454	0.024
Met-age (years) ^b^	66.421 ± 10.746	75.083 ± 7.973	<0.001	−0.961	67.440 ± 9.323	75.967 ± 8.628	<0.001	−0.953
Difference in age (DIA) (years) ^c^	12.157 ± 8.434	5.750 ± 12.337	0.044	0.572	13.680 ± 5.528	3.200 ± 13.039	0.001	1.014
Body-mass index (BMI) (kg/m^2^) ^b^	27.374 ± 5.437	30.731 ± 4.820	0.023	−0.666	26.412 ± 4.016	32.203 ± 4.702	<0.001	−1.315
Body composition								
Body fat (%) ^b^	21.763 ± 8.820	28.256 ± 8.912	0.013	−0.731	21.164 ± 6.520	30.053 ± 9.480	<0.001	−1.075
Muscle mass (kg) ^b^	23.253 ± 4.164	26.292 ± 5.047	0.029	−0.638	23.688 ± 4.795	26.537 ± 4.756	0.032	−0.597
Bone mass (kg) ^b^	2.105 ± 0.357	2.356 ± 0.440	0.038	−0.605	2.140 ± 0.411	2.377 ± 0.417	0.040	−0.571
Body water (%) ^b^	29.253 ± 4.687	33.275 ± 5.828	0.012	−0.736	29.444 ± 4.512	33.920 ± 5.941	0.003	−0.838
Visceral fat (%) ^b^	8.316 ± 2.964	11.167 ± 3.806	0.006	−0.805	7.920 ± 2.197	12.067 ± 3.787	<0.001	−1.309
Leg muscle mass Index ^b^	84.789 ± 8.935	77.000 ± 7.231	<0.001	0.992	84.320 ± 8.440	75.833 ± 6.773	<0.001	1.121
Metabolic rate ^b^	9.211 ± 2.485	7.111 ± 2.252	0.003	0.900	9.280 ± 2.011	6.633 ± 2.282	<0.001	1.224

Notes: Difference between groups were determined using (^a^) Chi-square test, (^b^) Student’s *t*-test, and (^c^) Mann–Whitney’s U-test.

**Table 2:** Pearson correlation matrix between chronological age, anthropometric parameters, body composition variables and Met-age.

	(1)	(2)	(3)	(4)	(5)	(6)	(7)	(8)
(1) Age								
(2) BMI	−0.257							
(3) Body fat	−0.287 *	0.910 ***						
(4) Muscle mass	−0.244	0.506 ***	0.405 **					
(5) Bone mass	−0.253	0.509 ***	0.411 **	0.998 ***				
(6) Body water	−0.324 *	0.528 ***	0.434 **	0.928 ***	0.925 ***			
(7) Visceral fat	−0.290 *	0.703 ***	0.653 ***	0.541 ***	0.536 ***	0.762 ***		
(8) Leg muscle mass index	0.206	−0.629 ***	−0.722 ***	−0.282 *	−0.279 *	−0.489 ***	−0.825 ***	
Met-age	0.293	0.516 ***	0.574 ***	0.132	0.121	0.251	0.581 ***	−0.644 ***

Notes: Pearson’s r correlation test: * *p* < 0.05, ** *p* < 0.01, *** *p* < 0.001.

## Data Availability

The data presented in this study are available upon request from the corresponding author due to ethical reasons.

## Abbreviations

The following abbreviations are used in this manuscript:

AUC	Area Under the Curve
BIA	Bioelectrical Impedance Analysis
BMI	Body Mass Index
BMR	Basal Metabolic Rate
CI	Confidence Interval
CVR	Cardiovascular Risk
CVD	Cardiovascular Disease
DIA	Difference between Chronological Age and Metabolic Age
HDL	High-Density Lipoprotein
LDA	Linear Discriminant Analysis
Met-age	Metabolic Age
MetS	Metabolic Syndrome
MNA	Mini Nutritional Assessment
OR	Odds Ratio
ROC	Receiver Operating Characteristic
SCORE2-OP	Systematic Coronary Risk Evaluation 2–Older Persons

## References

[1] Guo J., Huang X., Dou L., Yan M., Shen T., Tang W., Li J. (2022). Aging and Aging-Related Diseases: From Molecular Mechanisms to Interventions and Treatments. Signal Transduct. Target. Ther..

[2] World Health Organization Ageing and Health. https://www.who.int/news-room/fact-sheets/detail/ageing-and-health.

[3] Roth G.A., Mensah G.A., Johnson C.O., Addolorato G., Ammirati E., Baddour L.M., Barengo N.C., Beaton A., Benjamin E.J., Benziger C.P. (2020). Global Burden of Cardiovascular Diseases and Risk Factors, 1990–2019: Update From the GBD 2019 Study. J. Am. Coll. Cardiol..

[4] Dagenais G.R., Leong D.P., Rangarajan S., Lanas F., Lopez-Jaramillo P., Gupta R., Diaz R., Avezum A., Oliveira G.B.F., Wielgosz A. (2020). Variations in Common Diseases, Hospital Admissions, and Deaths in Middle-Aged Adults in 21 Countries from Five Continents (PURE): A Prospective Cohort Study. Lancet.

[5] Townsend N., Kazakiewicz D., Lucy Wright F., Timmis A., Huculeci R., Torbica A., Gale C.P., Achenbach S., Weidinger F., Vardas P. (2022). Epidemiology of Cardiovascular Disease in Europe. Nat. Rev. Cardiol..

[6] Fahed G., Aoun L., Zerdan M.B., Allam S., Zerdan M.B., Bouferraa Y., Assi H.I. (2022). Metabolic Syndrome: Updates on Pathophysiology and Management in 2021. Int. J. Mol. Sci..

[7] Saklayen M.G. (2018). The Global Epidemic of the Metabolic Syndrome. Curr. Hypertens. Rep..

[8] Neeland I.J., Lim S., Tchernof A., Gastaldelli A., Rangaswami J., Ndumele C.E., Powell-Wiley T.M., Després J.-P. (2024). Metabolic Syndrome. Nat. Rev. Dis. Primers.

[9] Zila-Velasque J.P., Grados-Espinoza P., Challapa-Mamani M.R., Sánchez-Alcántara F., Cedillo-Balcázar J., Aziz-Delgado C.S., Hernandez-Bustamante E.A., Tejada-Flores J., Piano Suárez A., Pacheco-Mendoza J. (2024). Prevalence of Metabolic Syndrome and Its Components According to Altitude Levels: A Systematic Review and Meta-Analysis. Sci. Rep..

[10] Deng Y., Yang Q., Hao C., Wang H.H., Ma T., Chen X., Ngai F.-W., Xie Y.J. (2025). Combined Lifestyle Factors and Metabolic Syndrome Risk: A Systematic Review and Meta-Analysis. Int. J. Obes..

[11] Kaptoge S., Pennells L., De Bacquer D., Cooney M.T., Kavousi M., Stevens G., Riley L.M., Savin S., Khan T., Altay S. (2019). World Health Organization Cardiovascular Disease Risk Charts: Revised Models to Estimate Risk in 21 Global Regions. Lancet Glob. Health.

[12] Hamczyk M.R., Nevado R.M., Barettino A., Fuster V., Andrés V. (2020). Biological Versus Chronological Aging: JACC Focus Seminar. J. Am. Coll. Cardiol..

[13] Espejo-Antúnez L., Fernández-Morales C., Moreno-Vázquez J.M., Tabla-Hinojosa F.B., Cardero-Durán M.d.l.Á., Albornoz-Cabello M. (2022). Assessment from a Biopsychosocial Approach of Flight-Related Neck Pain in Fighter Pilots of Spanish Air Force. An Observational Study. Diagnostics.

[14] Durán-Gómez N., Martín-Parrilla M.Á., Montanero-Fernández J., López-Jurado C.F., Rodríguez-Rivero L., Cáceres M.C. (2026). Patterns of Vulnerability: Frailty, Multimorbidity, and Physical Health-Related Quality of Life in Institutionalised Older Adults. Healthcare.

[15] Durán-Gómez N., Martín-Parrilla M.Á., Montanero-Fernández J., López-Jurado C.F., Pérez-Civantos D., Cáceres M.C. (2025). Impact of Mental Health Status and Clinical Factors on Health-Related Quality of Life in Patients Undergoing Haemodialysis: A Multicentre Cross-Sectional Study. BMJ Open.

[16] Zhao D., Wang Y., Wong N.D., Wang J. (2024). Impact of Aging on Cardiovascular Diseases: From Chronological Observation to Biological Insights: JACC Family Series. JACC Asia.

[17] Wang T., Beyene H.B., Yi C., Cinel M., Mellett N.A., Olshansky G., Meikle T.G., Wu J., Dakic A., Watts G.F. (2024). A Lipidomic Based Metabolic Age Score Captures Cardiometabolic Risk Independent of Chronological Age. EBioMedicine.

[18] Vásquez-Alvarez S., Bustamante-Villagomez S.K., Vazquez-Marroquin G., Porchia L.M., Pérez-Fuentes R., Torres-Rasgado E., Herrera-Fomperosa O., Montes-Arana I., Gonzalez-Mejia M.E. (2021). Metabolic Age, an Index Based on Basal Metabolic Rate, Can Predict Individuals That Are High Risk of Developing Metabolic Syndrome. High Blood Press. Cardiovasc. Prev..

[19] Ramírez Gallegos I., Marina Arroyo M., López-González Á.A., Vicente-Herrero M.T., Vallejos D., Sastre-Alzamora T., Ramírez-Manent J.I. (2024). The Effect of a Program to Improve Adherence to the Mediterranean Diet on Cardiometabolic Parameters in 7034 Spanish Workers. Nutrients.

[20] Parker H., Hunt E.T., Brazendale K., von Klinggraeff L., Jones A., Burkart S., Dugger R., Armstrong B., Beets M.W., Weaver R.G. (2022). Accuracy and Precision of Opportunistic Measures of Body Composition from the Tanita DC-430U. Child. Obes..

[21] Özderya A., Tekbil M., Ertürk N., Maz M.A., Yerlikaya M.G., Sayin M.R. (2024). Comparison of Coronary Artery Lesion Complexity and Body Component Parameters Measured by TANITA. Adv. Interv. Cardiol..

[22] Elguezabal-Rodelo R., Ochoa-Précoma R., Vazquez-Marroquin G., Porchia L.M., Montes-Arana I., Torres-Rasgado E., Méndez-Fernández E., Pérez-Fuentes R., Gonzalez-Mejia M.E. (2021). Metabolic Age Correlates Better than Chronological Age with Waist-to-Height Ratio, a Cardiovascular Risk Index. Med. Clin..

[23] Idrees T., Castro-Revoredo I.A., Oh H.D., Gavaller M.D., Zabala Z., Moreno E., Moazzami B., Galindo R.J., Vellanki P., Cabb E. (2024). Continuous Glucose Monitoring-Guided Insulin Administration in Long-Term Care Facilities: A Randomized Clinical Trial. J. Am. Med. Dir. Assoc..

[24] Khan M.A., Munshi M.N., Slyne C., Joyce N.R., Zullo A.R. (2025). Agreement Between Fingerstick Blood Glucose and Continuous Glucose Monitor Measures Among Long-Term Care Facility Residents. J. Am. Geriatr. Soc..

[25] von Elm E., Altman D.G., Egger M., Pocock S.J., Gøtzsche P.C., Vandenbroucke J.P. (2008). The Strengthening the Reporting of Observational Studies in Epidemiology (STROBE) Statement: Guidelines for Reporting Observational Studies. J. Clin. Epidemiol..

[26] Corporation T. (2020). MC-580M Body Composition Analyzer: Instruction Manual.

[27] (2021). SCORE2-OP working group and ESC Cardiovascular risk collaboration. SCORE2-OP Risk Prediction Algorithms: Estimating Incident Cardiovascular Event Risk in Older Persons in Four Geographical Risk Regions. Eur. Heart J..

[28] Alberti K.G.M.M., Eckel R.H., Grundy S.M., Zimmet P.Z., Cleeman J.I., Donato K.A., Fruchart J.C., James W.P.T., Loria C.M., Smith S.C. (2009). Harmonizing the Metabolic Syndrome: A Joint Interim Statement of the International Diabetes Federation Task Force on Epidemiology and Prevention; National Heart, Lung, and Blood Institute; American Heart Association; World Heart Federation; International Atherosclerosis Society; And International Association for the Study of Obesity. Circulation.

[29] Visseren F., Mach F., Smulders Y.M., Carballo D., Koskinas K.C., Bäck M., Benetos A., Biffi A., Boavida J.M., Capodanno D. (2021). 2021 ESC Guidelines on Cardiovascular Disease Prevention in Clinical Practice. Eur. Heart J..

[30] Lang P.O., Trivalle C., Vogel T., Proust J., Papazian J.P. (2015). Markers of Metabolic and Cardiovascular Health in Adults: Comparative Analysis of DEXA-Based Body Composition Components and BMI Categories. J. Cardiol..

[31] Blüher M. (2020). Metabolically Healthy Obesity. Endocr. Rev..

[32] Mazalin Protulipac J., Sonicki Z., Reiner Ž. (2015). Cardiovascular Disease (CVD) Risk Factors in Older Adults—Perception and Reality. Arch. Gerontol. Geriatr..

[33] Cruz-Jentoft A.J., Bahat G., Bauer J., Boirie Y., Bruyère O., Cederholm T., Cooper C., Landi F., Rolland Y., Sayer A.A. (2019). Sarcopenia: Revised European Consensus on Definition and Diagnosis. Age Ageing.

[34] Lee S.W., Youm Y., Lee W.J., Choi W., Chu S.H., Park Y.R., Kim H.C. (2015). Appendicular Skeletal Muscle Mass and Insulin Resistance in an Elderly Korean Population: The Korean Social Life, Health And Aging Project-Health Examination Cohort. Diabetes Metab. J..

[35] Kim K., Park S.M. (2018). Association of Muscle Mass and Fat Mass with Insulin Resistance and the Prevalence of Metabolic Syndrome in Korean Adults: A Cross-Sectional Study. Sci. Rep..

[36] Seko T., Akasaka H., Koyama M., Himuro N., Saitoh S., Miura T., Mori M., Ohnishi H. (2023). Preserved Lower Limb Muscle Mass Prevents Insulin Resistance Development in Nondiabetic Older Adults. J. Am. Med. Dir. Assoc..

[37] Jiang M., Ren X., Han L., Zheng X. (2024). Associations between Sarcopenic Obesity and Risk of Cardiovascular Disease: A Population-Based Cohort Study among Middle-Aged and Older Adults Using the CHARLS. Clin. Nutr..

[38] Farmer R.E., Mathur R., Schmidt A.F., Bhaskaran K., Fatemifar G., Eastwood S.V., Finan C., Denaxas S., Smeeth L., Chaturvedi N. (2019). Associations between Measures of Sarcopenic Obesity and Risk of Cardiovascular Disease and Mortality: A Cohort Study and Mendelian Randomization Analysis Using the UK Biobank. J. Am. Heart Assoc..

[39] Janssen I., Miller E., Dzongowski E., Miakisheva K., Ross R. (2022). A Compositional Analysis Study of Body Composition and Cardiometabolic Risk Factors. Obesity.

[40] Gradidge P.J.L., Norris S.A., Jaff N.G., Crowther N.J. (2016). Metabolic and Body Composition Risk Factors Associated with Metabolic Syndrome in a Cohort of Women with a High Prevalence of Cardiometabolic Disease. PLoS ONE.

[41] Siervo M., Lara J., Celis-Morales C., Vacca M., Oggioni C., Battezzati A., Leone A., Tagliabue A., Spadafranca A., Bertoli S. (2016). Age-Related Changes in Basal Substrate Oxidation and Visceral Adiposity and Their Association with Metabolic Syndrome. Eur. J. Nutr..

[42] Hudzik B., Nowak J., Szkodziński J., Zubelewicz-Szkodzińska B. (2021). Visceral Adiposity in Relation to Body Adiposity and Nutritional Status in Elderly Patients with Stable Coronary Artery Disease. Nutrients.

[43] Lee J.J., Pedley A., Hoffmann U., Massaro J.M., Levy D., Long M.T. (2018). Visceral and Intrahepatic Fat Are Associated with Cardiometabolic Risk Factors Above Other Ectopic Fat Depots: The Framingham Heart Study. Am. J. Med..

[44] Cesaro A., De Michele G., Fimiani F., Acerbo V., Scherillo G., Signore G., Rotolo F.P., Scialla F., Raucci G., Panico D. (2023). Visceral Adipose Tissue and Residual Cardiovascular Risk: A Pathological Link and New Therapeutic Options. Front. Cardiovasc. Med..

[45] Lee J.J., Pedley A., Hoffmann U., Massaro J.M., Keaney J.F., Vasan R.S., Fox C.S. (2015). Cross-Sectional Associations of Computed Tomography (CT)-Derived Adipose Tissue Density and Adipokines: The Framingham Heart Study. J. Am. Heart Assoc..

